# Distinct afferent innervation patterns within the human proximal and distal esophageal mucosa

**DOI:** 10.1152/ajpgi.00175.2014

**Published:** 2015-01-08

**Authors:** Philip Woodland, Rubina Aktar, Engelbert Mthunzi, Chung Lee, Madusha Peiris, Sean L. Preston, L. Ashley Blackshaw, Daniel Sifrim

**Affiliations:** Barts and the London School of Medicine and Dentistry, Queen Mary University of London, London, United Kingdom

**Keywords:** gastroesophageal reflux, mucosal integrity, impedance, afferent neurons, peripheral sensitization

## Abstract

Little is known about the mucosal phenotype of the proximal human esophagus. There is evidence to suggest that the proximal esophagus is more sensitive to chemical and mechanical stimulation compared with the distal. This may have physiological relevance (e.g., in prevention of aspiration of gastroesophageal refluxate), but also pathological relevance (e.g., in reflux perception or dysphagia). Reasons for this increased sensitivity are unclear but may include impairment in mucosal barrier integrity or changes in sensory innervation. We assessed mucosal barrier integrity and afferent nerve distribution in the proximal and distal esophagus of healthy human volunteers. In 10 healthy volunteers baseline proximal and distal esophageal impedance was measured in vivo. Esophageal mucosal biopsies from the distal and proximal esophagus were taken, and baseline transepithelial electrical resistance (TER) was measured in Ussing chambers. Biopsies were examined immunohistochemically for presence and location of calcitonin gene-related peptide (CGRP)-immunoreactive nerve fibers. In a further four healthy volunteers we investigated for colocalization of CGRP and protein gene product (PGP) 9.5 immunoreactivity in nerve fibers. Baseline impedance was higher in the proximal than in the distal esophagus [2,936 Ω (SD578) vs. 2,229 Ω (SD821); *P* = 0.03], however, baseline TER was not significantly different between them. Mucosal CGRP-immunoreactive nerves were found in the epithelium of both proximal and distal esophagus, but were located more superficially in the proximal mucosa compared with the distal [11.5 (SD7) vs. 21.7 (SD5) cell layers from lumen, *P* = 0.002] 19% of proximal, and 10% of distal mucosal PGP-immunoreactive fibers colocalized with CGRP. PGP-immunoreactive fibers were also significantly closer to the luminal surface in the proximal compared with the distal esophagus (*P* < 0.001). We conclude that mucosal barrier integrity is similar in proximal and distal esophagus, but proximal mucosal afferent nerves are in a more superficial location. The enhanced sensitivity to reflux-evoked symptoms of the proximal esophagus most likely has an anatomical basis.

historically, the distal esophagus has been the focus of investigation into pathogenesis of esophageal sensation in disease [such as in gastroesophageal reflux disease (GERD)]. However, there is some experimental evidence to suggest that the proximal esophagus may play an important sensory role that is likely to be relevant in both health and disease. The proximal esophagus appears to be more sensitive than the distal to mechanical distension (15) and electrical stimuli than the distal. There is also evidence that the proximal esophagus is more sensitive than the distal esophagus to acid-evoked pain during experimental perfusion (19, 24).

Physiologically, it can be hypothesized that heightened sensitivity of the proximal esophagus to luminal contents may have a protective role. Gastroesophageal reflux is a daily event, even in health (22). Whereas distal reflux events can be symptomatic (and prolonged distal esophageal acidification can result in significant complications ranging from erosions to adenocarcinoma), reflux events reaching the proximal esophagus increase the risk of aspiration of gastric contents into the airways. It is therefore likely that a heightened perception of reflux in the proximal esophagus is needed to initiate both conscious (swallowing) and reflex protective mechanisms (secondary peristalsis and esophago-sphincteric reflexes) that are in part acid mediated, and that result in an increase in upper esophageal sphincter pressure (9, 27).

In pathological situations, there is evidence that sensation in the proximal esophagus may be of importance. GERD affects 10–20% of the Western population (4, 6). Reflux monitoring using multiple pH sensors or combined pH-impedance has enabled characterization of not only the pH but also the proximal extent of reflux events. In patients with GERD, reflux events reaching the proximal esophagus are more likely to be perceived than those reaching only the distal esophagus (3, 32). Furthermore, in GERD patients refractory to proton pump inhibitor (PPI), impedance-pH studies “on” therapy have indicated that a high proximal extent is the most important factor in determining whether or not a reflux episode will be perceived by the patient (28, 36).

The mechanism of the relative increased sensitivity of the proximal esophagus is unknown. It is possible that it may result from a mucosal factor. One possibility is that it is due to a weaker barrier function of the mucosa in the proximal esophagus. It is known that differences in permeability of the distal esophageal mucosa can be important in pathogenesis of reflux disease, whereby a weaker mucosa may allow greater access of noxious intraluminal components to nociceptive afferent fibers. Such impairment of mucosal integrity can also be demonstrated functionally ex vivo and in vivo (35). In human biopsies, the impaired barrier function can be expressed in terms of a low mucosal electrical resistance [transepithelial electrical resistance (TER)] or permeability (8, 25, 26), and, with the use of in vivo impedance techniques (which in the absence of bolus transit are able to measure esophageal transmucosal impedance), it can be expressed in terms of a low mucosal basal impedance (13, 23, 33). In the empty collapsed esophagus the mucosa lies against the impedance catheter and offers impedance to current flow between impedance electrode pairs. It has been shown that low impedance is associated with impaired mucosal barrier integrity in the esophagus (7, 33). Subjects who perceive an acid challenge as heartburn have a lower baseline distal esophageal impedance than those who do not (33).

It is also possible that the enhanced sensitivity of the proximal esophagus is related to a distinct sensory innervation. Although there is some data about the distal esophageal innervation in health and in reflux disease (18), and some animal data supporting an unequal innervation throughout the esophagus [in the rat, density of nerve fibers is most prominent in the upper cervical region of the esophagus, and decreases in the lower cervical and thoracic esophagus (5, 17, 29)], data to compare mucosal innervation in the proximal and distal human esophagus are lacking. Further insight into this may be of physiological and pathological importance, and a differential distribution of sensory afferent fibers in the human esophageal mucosa may contribute to special differences in esophageal sensitivity.

To understand potential mechanisms for the relative heightened proximal esophageal sensitivity, the aim of this study was to characterize proximal esophageal mucosal integrity and afferent innervation in healthy human subjects.

## METHODS

### Subjects

Fourteen asymptomatic volunteers (mean age 24 yr, range 21–42) were studied. None had a history of esophageal symptoms, and none was taking medications for gastrointestinal problems or analgesics.

All had normal esophageal appearance on endoscopy.

The study was granted ethical approval by East London and the City Research Ethics Committee (07/H0705/57), and all subjects signed a written consent form.

### In Vivo Study

#### Procedure.

This was performed in 10 healthy volunteers. An intraluminal combined pH-impedance catheter was used to measure baseline esophageal mucosal impedance. The lower esophageal sphincter position was located using high-resolution manometry. After esophageal manometry, the pH-impedance catheter was lubricated and passed transnasally into the esophagus such that the pH sensor was placed 5 cm above the manometrically defined lower esophageal sphincter. Baseline distal esophageal impedance was measured at 3 cm above the lower esophageal sphincter. Baseline proximal impedance was measured at 17 cm above the lower esophageal sphincter. The data were recorded on a portable digital data logger (Sandhill Scientific) and analyzed on proprietary pH-impedance analysis software (Bioview Analysis, Sandhill Scientific).

After placement of the pH-impedance catheter a baseline proximal esophageal impedance measurement (in Ω) was made with the subject in an upright sitting position for 15 min. The baseline proximal and distal esophageal impedance was calculated as the average impedance between 5 and 15 min after catheter placement. Swallows and reflux episodes (if any) were excluded from baseline analysis.

### In Vitro Studies

#### Endoscopy.

Endoscopic procedures were performed using pharyngeal local anesthetic spray. In each subject four esophageal mucosal biopsies were taken (Radial Jaw 3 forceps; Boston Scientific), two from 20 cm above the squamo-columnar junction (proximal esophageal biopsies) and two from 3 cm above the squamo-columnar junction (distal esophageal biopsies). Biopsies were carefully orientated immediately and placed on cellular acetate paper. One proximal and one distal biopsy were placed into a preoxygenated Krebs-Henseleit buffer solution at pH 7.4 and at 4°C and were then rapidly transported to the laboratory for Ussing chamber experiments. The other proximal and distal biopsies were placed immediately in 4% paraformaldehyde for subsequent histological evaluation.

### Ussing Chamber Studies

Biopsies from 14 volunteers were orientated and mounted in Ussing chambers (Mussler Scientific Instruments, Aachen, Germany), as we have previously described (34). Immediately on mounting the biopsies were bathed on both luminal and basal sides with Krebs-Henseleit buffer at pH 7.4, 37°C, and the solution was continuously bubbled with carbogen gas. After making a correction for fluid and circuit resistance, basal TER (in Ω·cm^2^) was calculated according to Ohm's law from the voltage deflections induced by bipolar current pulses of 50 μA, duration 200 ms every 6 s applied through platinum wires. All experiments were conducted in open-circuit conditions. The system was equilibrated at 37°C until a stable TER baseline was established (typically 20 min).

Biopsies would be deemed suitable for Ussing chamber study if: *1*) the biopsy could be clearly seen to cover the 1.5-mm aperture on stereomicroscopy (to ensure no visible potential for leak), *2*) baseline TER was >50 Ω·cm^2^, and *3*) there was no visible fluorescein cross-chamber leakage after 20 min when applied at a concentration of 1 mg/ml to one chamber at the end of the experiment.

### Immunohistochemical Studies

Proximal and distal esophageal biopsies were fixed in 4% paraformaldehyde overnight, followed by cryoprotection in 30% sucrose in phosphate-buffered saline (PBS) for 24 h at 4^0^C, followed by 30% sucrose PBS-optimum cutting temperature compound (1:1) at 4^0^C. Sections (10 μm) were cut on a cryostat and mounted on positive-charged glass slides. Sections were air-dried for 1 h, serum blocked to prevent nonspecific binding, and incubated with a primary antibody to calcitonin gene-related peptide [CGRP, used as a marker of nociceptive sensory innervation (10)], 1:500 (monoclonal mouse anti-human, Pierce Antibodies ABS 026-05-02) at 4^0^C overnight. Sections were then washed three times for 10 min with PBS-0.2% Triton, and secondary antibody was applied (donkey anti-mouse 488; Invitrogen) and incubated for 1 h. Sections were then washed with PBS-0.2% Triton 3 × 10 min and mounted with HardSet Vectashield. Negative controls were prepared with the primary antibody omitted, and showed no labeling. Fluorescence was visualized using an epifluorescent microscope (Olympus BX61). All images were obtained with a ×40 oil immersion lens under the 488-nm excitation setting.

Adapting a methodology previously used to assess human corneal innervation, we studied the location of afferent nerve fibers in the human esophageal mucosa (16). In 10 volunteers CGRP-immunoreactive fibers were identified within the squamous epithelium. In each section, the position of the most superficial fiber (relative to the luminal surface of the section) was recorded in terms of number of cell layers from the fiber to the luminal surface.

In a further four volunteers colocalization of CGRP and protein gene product 9.5 (PGP, Dako: monoclonal rabbit anti-human PGP protein clone 2F11, dilution 1 in 2,000) was performed to confirm that neural structures were being labeled. In this subset of volunteers we also measured distance from the luminal surface of fibers in terms of distance (μm) to ensure that the method of measurement did not alter the results.

We decided to primarily investigate the nerve fiber distance from the luminal aspect of the biopsy since many papers demonstrate functionally that the proximity of an ending to a structure (or the lumen) correlates with its likelihood of responding to stimuli delivered to that structure.

Investigators performing the in vitro studies were blinded as to the nature (proximal or distal) of the biopsy specimen. Accurate quantification of the number of fibers could not be performed because serpiginous single fibers were often seen in multiple 10-μm sections, or could appear several times in the same section. Acknowledging this limitation, we estimated density of mucosal fibers in the subset of four volunteers. Five fields of view (1.44 megapixel) were randomly obtained with a QImaging camera and analyzed with ImageJ for total number of immunoreactive pixels in the region of interest within the mucosal layer.

### Statistical Analysis

Data are presented as means and standard deviation (SD). Baseline impedance in each subject was compared using a paired *t*-test. Baseline TER and change in TER on exposure to acidic solution between proximal and distal esophageal biopsies from the same subject were tested with a paired *t*-test.

For each subject the distance of each visualized fiber from the luminal biopsy surface was recorded, as was the median distance per subject. These distances were compared by paired *t*-test for paired specimens (upper and lower esophagus from the same subject).

## RESULTS

### In Vivo Study

#### Baseline proximal and distal impedance.

**Fig. 1.**
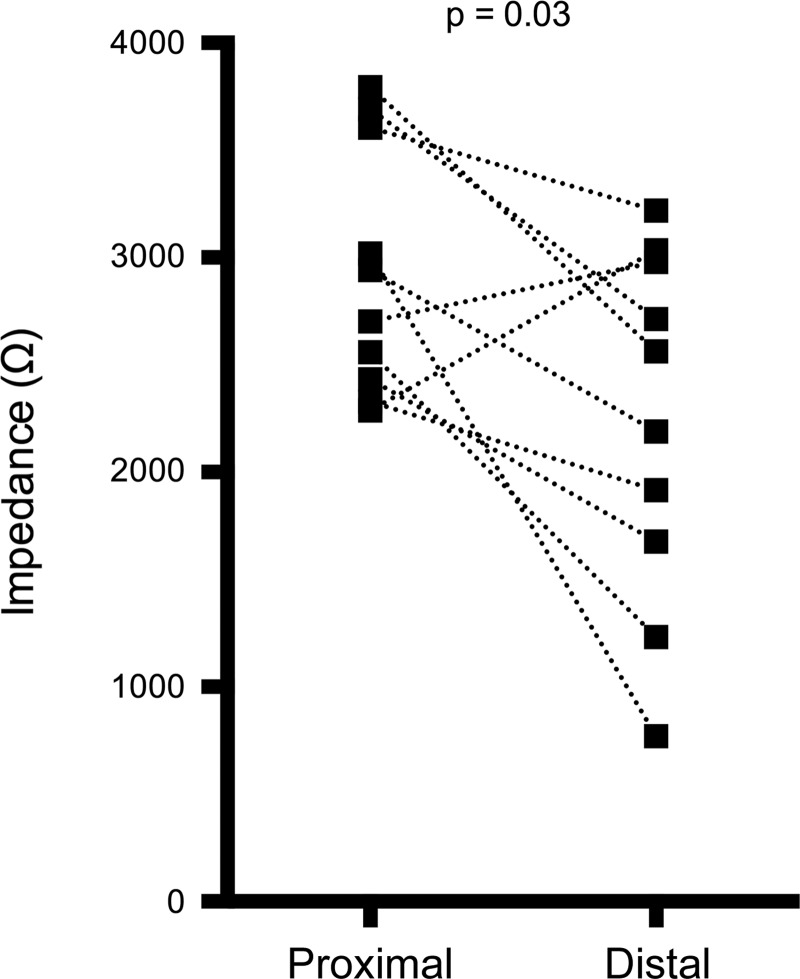
Baseline impedance in the distal and proximal esophagus. Paired values connected by line.

Baseline proximal esophageal impedance was found to be significantly higher than baseline distal impedance [2,936 Ω (SD578) vs. 2,229 Ω (SD821); *P* = 0.03, Fig. 1].

### In Vitro Studies

#### Baseline TER.

**Fig. 2.**
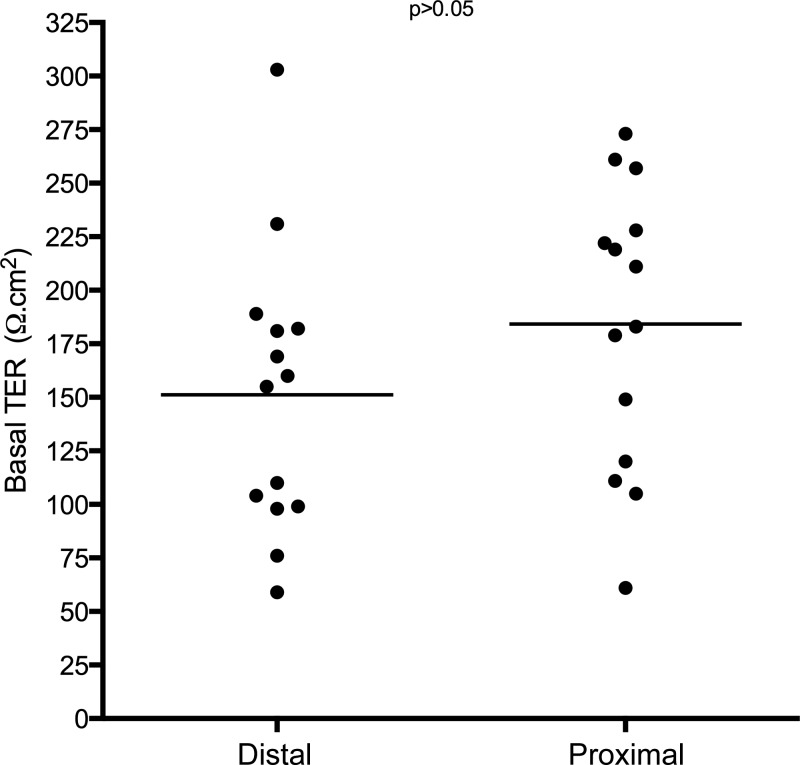
Transepithelial electrical resistance (TER) in biopsies from the proximal and distal esophagus.

All biopsies were deemed to be suitable for Ussing chamber experiments. The mean basal TER in the proximal esophagus was 184.2 Ω·cm^2^ (SD66). In the distal esophagus the mean TER was 151.1 Ω·cm^2^ (SD66). There was no significant difference between these values (Fig. 2).

#### Comparison of innervation of the proximal and distal esophagus.

CGRP-immunoreactive fibers could be seen within the epithelium of at least one section from each biopsy taken from the proximal and distal esophagus. Neural origin was confirmed with staining for the pan-neuronal marker PGP. Nineteen percent of proximal and 10% of distal PGP-immunoreactive fibers colocalized with CGRP.

**Fig. 3.**
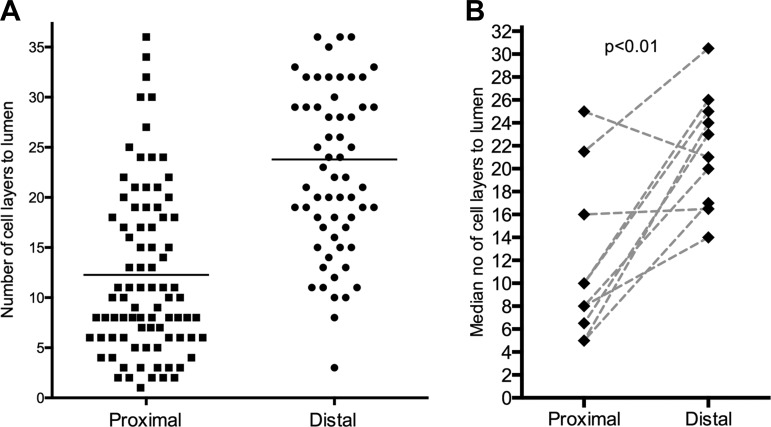
Comparison of location of calcitonin gene-related peptide (CGRP)-immunoreactive nerve fibers (expressed as no. of cells from the luminal surface of the epithelium) in the proximal and distal esophageal. *A*: location of all visualized esophageal fibers in all examined sections. *B*: the median location per subject is shown, with lines joining paired results from the same subject.

**Fig. 4.**
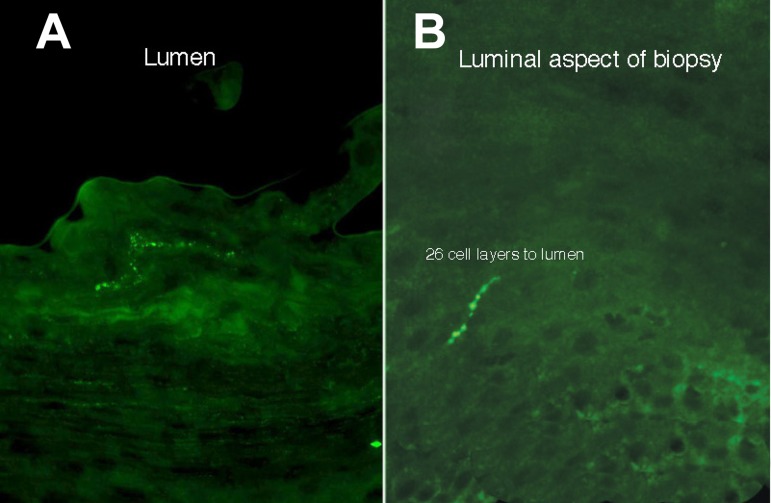
Transverse sections of esophageal biopsies showing CGRP-immunoreactivity (lumen uppermost). *A*: example of a proximal esophageal biopsy, with CGRP-immunoreactive fibers a few cell layers from the luminal surface. *B*: example of a distal esophageal biopsy, demonstrating a nerve fiber in the basal epithelium (most superficial part of fiber 26 cell layers from the lumen). Images magnified ×40. Scale bar represents 100 μm.

CGRP-immunoreactive nerves were significantly more superficial in the proximal esophageal biopsies than in the distal [11.5 (SD7) vs. 21.7 (SD5) cell layers from the lumen, *P* < 0.01; Fig. 3*B*]. Example images are seen in Fig. 4.

When measured in terms of micrometers, both CGRP- and PGP-immunoreactive fibers were significantly closer to the luminal surface in the proximal mucosa compared with the distal mucosa [29.4 μm (SD31) vs. 187.5 μm (SD183), *P* < 0.0001, and 51.9 μm (SD36) vs. 199.2 μm (SD110), *P* < 0.0001, respectively].

There was no significant difference in density as measured by number of immunoreactive pixels in the proximal and distal esophagus [2,593 pixels (SD2,673) vs. 3,910 pixels (SD5,864), *P* = 0.43].

**Fig. 5.**
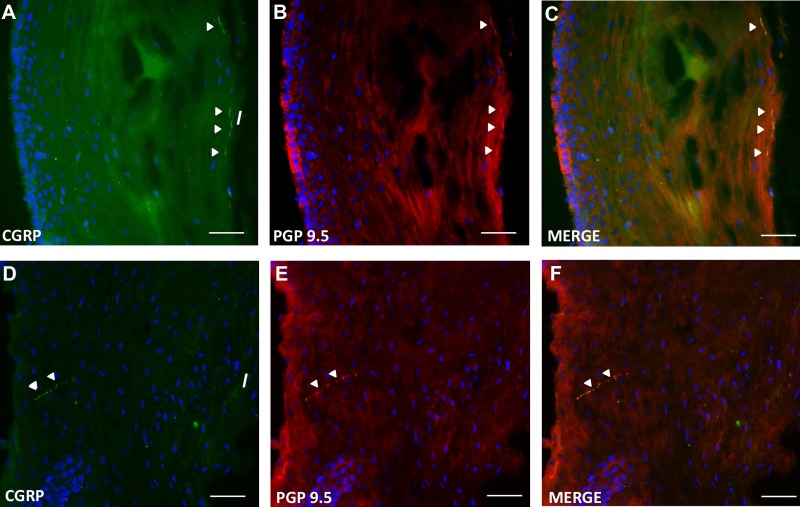
Representative immunostaining for CGRP and protein gene product (PGP) in the proximal (*A–C*) and distal (*D–F*) esophageal mucosa.

There are several features of the CGRP labeling that we saw that are important to note from a technical viewpoint and the rigor of our approach. *1*) The same fiber labeling is seen with PGP and CGRP (Fig. 5), confirming neuronal origin, and there was never any cellular label. We did not see any CGRP-immunoreactive fibers that were not also PGP-immunoreactive. *2*) With variable focus we could follow fibers back from the superficial layers to the deeper layers (data not shown), indicating they are not disconnected from the parent axon, and we see both types of ending (deep and superficial) in some sections of proximal esophagus. *3*) Many superficial regions in proximal esophagus were CGRP negative, indicating these endings, where they do occur, are specialized. *4*) The same CGRP antibody also labeled nerve fibers in human colon sections and whole mounts (data not shown). *5*) All control images were negative.

## DISCUSSION

This study provides a novel insight into the sensory contrast between the proximal and distal esophageal mucosa. This provides a potential mechanism for both the physiological protective function of increased sensitivity of the proximal esophagus and may also provide a platform for understanding the important sensory role of the proximal esophagus in disease. It is known that GERD patients are more sensitive to reflux events that reach the proximal esophagus compared with those restricted to the distal part (2, 28, 31). There is also supportive evidence to suggest that the proximal esophagus is more chemosensitive than the distal (19, 24). Our results suggest a likely mechanism of such regional sensitivity.

Our study showed: *1*) impaired mucosal barrier integrity of the proximal esophagus is unlikely to be the reason for proximal hypersensitivity because *a*) in vivo there is a greater baseline impedance in the proximal esophagus compared with the distal, and *b*) in vitro there is a trend toward higher baseline esophageal mucosal TER. *2*) In contrast with the relative robustness of proximal esophageal mucosal integrity, the location of mucosal peptidergic sensory fibers was closer to the lumen in the proximal esophagus compared with the distal.

There is a paucity of data available about the mucosal innervation of the proximal esophagus in humans. The need to gain understanding has been reinforced in recent years by the demonstration that the proximal esophagus appears to be more sensitive to chemical (19, 24), electrical, and mechanical (15) stimuli than the distal, and also by the realization that the proximal esophagus appears to play a greater role in esophageal disease symptoms than previously thought.

Esophageal sensation is likely to have a multifactorial basis. Afferent innervation from chemo- and mechanosensitive fibers and spinobulbar and cerebral processing of signals are all very important factors. Furthermore, a peripheral mucosal barrier function is considered to have a significant pathophysiological role (20). In previous experiments it has been demonstrated that ex vivo baseline TER (12) and in vivo impedance (13, 33) are lower in patients with GERD than in control subjects. We originally hypothesized that an enhanced proximal esophageal perception of acid may be a phenomenon related to altered proximal mucosal integrity. The findings of this study suggest that this is not the case. In basal conditions the integrity of the proximal mucosa appears stronger than the distal (with a significantly higher baseline impedance, and a trend toward a higher baseline TER). It is noted that the TER demonstrates significant interindividual variability. This has been demonstrated in previous studies (12, 30) and may be a reflection of technical difficulties in the test (for example, the trauma caused by biopsy procedure, manipulation to place in Ussing chamber, edge effect from the small aperture).

It appears that the enhanced sensitivity of the proximal esophagus is because of a different mechanism. We hypothesized that the mucosa of the proximal esophagus might have a distinct distribution of sensory nerves. We examined biopsies from the distal and proximal esophagus for the presence of mucosal sensory afferent fibers. We found that the location of these mucosal fibers was much closer to the esophageal lumen in the proximal esophagus compared with the distal. To our knowledge, this is the first time that the distribution of human esophageal mucosal sensory innervation has been investigated directly. We were primarily interested in CGRP-immunoreactive afferent fibers since we were particularly interested in nociceptive potential. In animal studies proximal esophageal mucosal fibers staining for CGRP have been shown often to be vagal (29). There is increasing evidence that even these vagal nerves may have a role in nociception (14), and as such the results may be relevant to symptom perception in human GERD. Our results in human esophageal mucosa suggest that the staining was nerve specific since in all cases there was colocalization with PGP 9.5. In fact, PGP-immunoreactive fibers were also significantly closer to the luminal aspect of the biopsy in the proximal esophagus. There were approximately five times as many PGP-immunoreactive fibers than CGRP-immunoreactive fibers, suggesting the presence of a subpopulation of CGRP-containing afferents. Whether or not these subpopulations have different functional roles remains to be fully investigated.

The density of innervation of the epithelium was not different between proximal and distal regions, but the striking feature was the proximity to the lumen. We considered it relevant to measure the number of layers of intercellular junctions the fibers are from the lumen since this more accurately reflects the physiological barrier to diffusion (1, 11, 12). However, the difference persists whether number of cell layers or distance in micrometers is calculated.

It is possible that nociception from proximal reflux events is due to the summative effect of simultaneous stimulation of both the distal and proximal esophagus. Our results also suggest that there may be a specialist innervation of the proximal esophageal mucosa to contribute. The superficial distribution of the proximal mucosal afferent fibers may constitute the sensory component of a defensive mechanism against airway aspiration facilitated by proximal reflux events (21). The more superficial distribution may also allow greater access of noxious gastroesophageal refluxate to afferent nerves and provide a mechanism to the observed enhanced perception of reflux events. It should be noted that this study does not definitively indicate a causal relationship between superficial mucosal nerve distribution and proximal sensitivity in NERD, since the study was done on healthy volunteers. Further work will need to be done to extrapolate these findings to a disease population. We would, however, expect the more superficial distribution of proximal nerves to persist in patients with reflux disease, and we have preliminary findings to suggest this. This superficial nature may render the nerves amenable to topical therapies aimed at reducing their sensitivity. Such a strategy could have potential benefit in GERD (including PPI-refractory disease) and proximal dysphagia symptoms.

In summary, this study suggests that proximal esophageal mucosal integrity is not impaired compared with that in the distal esophagus. As such, it appears that impairment in basal mucosal integrity does not underlie the clinical finding of increased proximal esophageal sensitivity. Perhaps underlying this differential sensitivity is a more superficial location of the nerve fibers in the proximal esophagus. New therapeutic strategies might target proximal esophageal hypersensitivity using topical mucosal protection to reduce potential contact of the refluxate with superficial nerves and/or pharmacological modulation of afferent signaling.

## GRANTS

A. Blackshaw is supported by a Wellcome Trust University Award.

## DISCLOSURES

Daniel Sifrim receives research grants from Sandhill Scientific, CO (USA) and Reckitt Benckiser, Hull (UK).

## AUTHOR CONTRIBUTIONS

Author contributions: P.W., L.A.B., and D.S. conception and design of research; P.W., R.A., E.M., C.L., and S.L.P. performed experiments; P.W., R.A., E.M., C.L., M.P., and L.A.B. analyzed data; P.W., M.P., L.A.B., and D.S. interpreted results of experiments; P.W. prepared figures; P.W. drafted manuscript; P.W., L.A.B., and D.S. edited and revised manuscript; D.S. approved final version of manuscript.
